# Mitochondria-Targeted Curcumin: A Potent Antibacterial Agent against Methicillin-Resistant *Staphylococcus aureus* with a Possible Intracellular ROS Accumulation as the Mechanism of Action

**DOI:** 10.3390/antibiotics12020401

**Published:** 2023-02-16

**Authors:** Carmen-Ecaterina Leferman, Laura Stoica, Bogdan Alexandru Stoica, Alin Dumitru Ciubotaru, Aida Corina Badescu, Camelia-Margareta Bogdanici, Tiberiu Paul Neagu, Cristina-Mihaela Ghiciuc

**Affiliations:** 1 Department of Pharmacology, Medical Specialties II, “Grigore T. Popa” University of Medicine and Pharmacy, 700115 Iasi, Romania; 2 Department of Ophthalmology, “Grigore T. Popa” University of Medicine and Pharmacy, 700115 Iasi, Romania; 3 Department of Cell and Molecular Biology, “Grigore T. Popa” University of Medicine and Pharmacy, 700115 Iasi, Romania; 4 Department of Biochemistry, “Grigore T. Popa” University of Medicine and Pharmacy, 700115 Iasi, Romania; 5 Department of Neurology, “Grigore T. Popa” University of Medicine and Pharmacy, 700115 Iasi, Romania; 6 Department of Microbiology (Bacteriology, Virology) and Parasitology, “Grigore T. Popa” University of Medicine and Pharmacy, 700115 Iasi, Romania; 7 Clinical Department No. 11—Plastic, Aesthetic and Reconstructive Microsurgery, “Carol Davila” University of Medicine and Pharmacy, 050474 Bucharest, Romania

**Keywords:** mitocurcumin, methicillin-resistant *Staphylococcus aureus*, redox mechanism, curcumin, triphenylphosphonium derivatives

## Abstract

Mitocurcumin (a triphenylphosphonium curcumin derivative) was previously reported as a selective antitumoral compound on different cellular lines, as well as a potent bactericidal candidate. In this study, the same compound showed strong antimicrobial efficacy against different strains of methicillin-resistant *Staphylococcus aureus* (MRSA). The minimum inhibitory concentration was identical for all tested strains (four strains of MRSA and one strain of methicillin-sensitive *Staphylococcus aureus*), suggesting a new mechanism of action compared with usual antibacterial agents. All tested strains showed a significant sensitivity in the low micromolar range for the curcumin-triphenylphosphonium derivative. This susceptibility was modulated by the menadione/glutathione addition (the addition of glutathione resulted in a significant increase in minimal inhibitory concentration from 1.95 to 3.9 uM, whereas adding menadione resulted in a decrease of 0.49 uM). The fluorescence microscopy showed a better intrabacterial accumulation for the new curcumin-triphenylphosphonium derivative compared with simple curcumin. The MitoTracker staining showed an accumulation of reactive oxygen species (ROS) for a *S. pombe* superoxide dismutase deleted model. All results suggest a new mechanism of action which is not influenced by the acquired resistance of MRSA. The most plausible mechanism is reactive oxygen species (ROS) overproduction after a massive intracellular accumulation of the curcumin-triphenylphosphonium derivative.

## 1. Introduction

In recent years, resistance to antibiotics has become a major world medical threat; hence, research for new antibiotics is more prolific than ever. A large number of studies are focused on enhancing the existent therapeutic molecules [1,2,3], but another possible approach is to discover and develop completely new classes of antimicrobial agents [4,5,6]. This study belongs to the second approach, unifying two ideas in one molecule: a well-known compound isolated from plants (curcumin) coupled with a special lipophilic, positively-charged radical (the triphenylposphonium radical).

Curcumin is a naturally occurring yellow pigment produced by the turmeric plant. It is commonly used as a spice in various culinary dishes and has a long history of use in traditional medicine, particularly in India and China. Over the last few decades, curcumin has gained increasing attention from the scientific community due to its potential antimicrobial properties.

The beneficial effects of curcumin are well documented in the current medical literature. As such, curcumin is one of the phytotherapeutic compounds which is being studied extensively worldwide, with various trials focused on several research areas, such as antitumoral, neuroprotective, hypocholesterolemic, antidiabetic, anti-inflammatory, antimicrobial effects, etc. [4,7,8,9,10,11].

Curcumin alone has been extensively investigated as an antimicrobial agent against a large range of bacterial strains, with promising results [12,13]. It is also an active compound which can improve the condition of sepsis due to its anti-inflammatory and antioxidant properties [14].

The antimicrobial activity has been attributed to its ability to interfere with the vital processes of these pathogens, such as cell division, protein synthesis, and cellular respiration [13]. Notably, several strains of antibiotic-resistant bacteria, including methicillin-resistant Staphylococcus aureus (MRSA), are effectively treated with curcumin [14].

Beyond its direct antimicrobial effects, curcumin has also been shown to enhance the efficacy of antibiotics [13]. By increasing the permeability of the bacterial cell wall, curcumin enhances the uptake of antibiotics, making them more effective against pathogens [15]. This combination therapy has been shown to be particularly effective against multi-drug-resistant bacteria, making curcumin a promising alternative to traditional antibiotics [15]. Curcumin has proven to have a variety of other health benefits, including antioxidant, anti-inflammatory, and anti-cancer properties [15].

Despite the promising results of these studies, it is important to note that more research is needed to fully understand the mechanisms underlying the antimicrobial activity of curcumin and to determine the optimal doses and administration methods. Nonetheless, the available evidence suggests that curcumin may represent a promising alternative to traditional antibiotics and may play a role in addressing the growing problem of antibiotic resistance.

A major problem encountered in various curcumin clinical trials lies in its low bioavailability, which leads to low plasma and tissue levels and hence a limitation in the therapeutic efficacy [16,17]. In addition, some studies reported relatively high levels of curcumin as the minimum inhibitory concentration in their experiments [12,18]. The best results for curcumin in the antimicrobial field were obtained in association with other antibiotics, when a synergic effect was obtained [18,19].

The triphenylphosphonium radical is known mainly because of its mitochondria-targeting properties, which generated a new class of special compounds able to accumulate in mitochondria, named mito-cans [20]. These highly targeted compounds exhibit an interesting range of therapeutic benefits, such as antioxidant properties [21], selective cytotoxicity [22] and recently were tested for antibacterial effects [23]. Regarding the antibacterial effects of triphenylphosphonium derivatives, it seems that the phylogenetic similarities between bacteria and mitochondrion leads to bacterial bioenergetic impairment, modifying the membrane potential [24].

Mitocurcumin is increasingly regarded as a potential antimicrobial agent. Mitochondria are a key target for many pathological conditions, as they play a critical role in regulating the host cell’s energy supply. In this way, mitocurcumin could also exert beneficial effects by interfering with mitochondrial metabolic processes that can be affected in severe infectious diseases.

The aim of this study was to test and to try to explain the antibacterial properties of mitocurcumin (MitoC, a triphenylphosphonium derivative of curcumin, which seeks to associate two beneficial pharmaceutical effects) against different strains of *Staphylococcus aureus*, especially the methicillin-resistant strains. The potential mechanism of action was investigated using fluorescence microscopy on bacteria and also on a eukaryotic model (*S. pombe* with a deleted superoxide dismutase gene). This model was chosen to illustrate the accumulation of ROS, due to the fact that our strain has a deletion which codes for one of the most important antioxidant enzymatic molecules present in both prokaryotes and eukaryotes. Using prooxidant/antioxidant compound associations, we were able to clarify the potential redox implications in the mitocurcumin antibacterial mechanism of action.

## 2. Results

### 2.1. The Antibacterial Effect of Mitocurcumin Is Significantly Higher Than Curcumin

**Figure 1:**
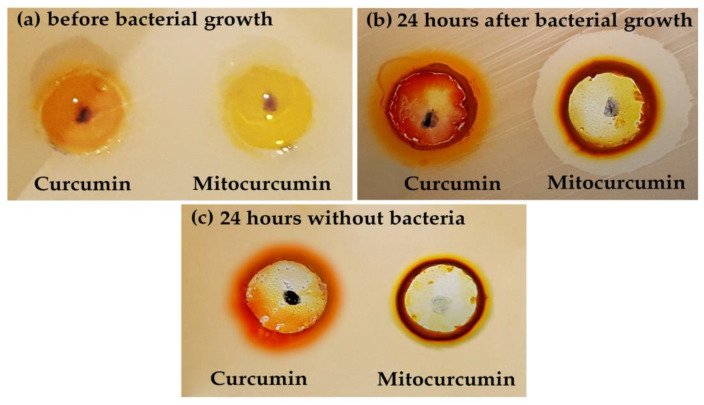
The well diffusion method showing the antibacterial effect of mitocurcumin against a standard MRSA strain (ATCC 700698) compared with curcumin at 10 µM (**a**) before and (**b**) after 24 h of incubation at 37 °C, and (**c**) control group with no bacteria inoculated on the agar plate.

Both the agar diffusion test and broth dilution assay showed a higher antimicrobial susceptibility for mitocurcumin if it is compared with simple curcumin. As depicted in Figure 1, at 10 µM the well diffusion method showed an important antibacterial efficiency for mitocurcumin (the inhibition diameter obtained in mm was 17 ± 1) against a standard MRSA strain (ATCC 700698), while curcumin displayed no effect. Table 1 showed that the inhibition diameters for the tested compounds were consistent with published literature for curcumin [12] and significantly increased for mitocurcumin. It is also noteworthy that while curcumin had a significantly improved antimicrobial effect after 10 min of visible light irradiation (1 J/cm^2^), the same procedure for mitocurcumin showed no effect (data not shown).

Interestingly, the antibacterial proprieties of mitocurcumin were the same for all tested strains. These results were obtained after the broth dilution assay, using serial dilutions (Table 2). Regardless of the strain type, both assays revealed the same values with an important difference between curcumin and mitocurcumin.

### 2.2. Both Compounds Generated Bacteria Fluorescence but Mitocurcumin Displayed a Stronger Effect

**Figure 2:**
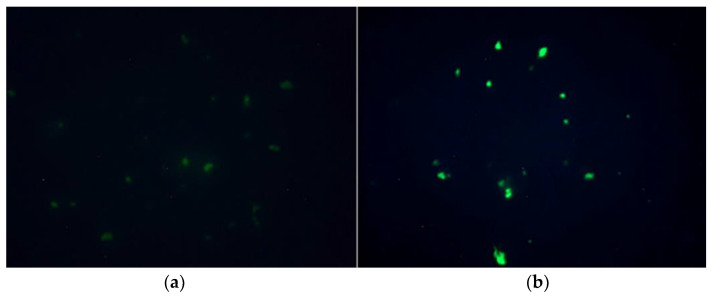
Fluorescence microscopy for (**a**) curcumin- and (**b**) mitocurcumin-treated bacteria.

**Figure 3:**
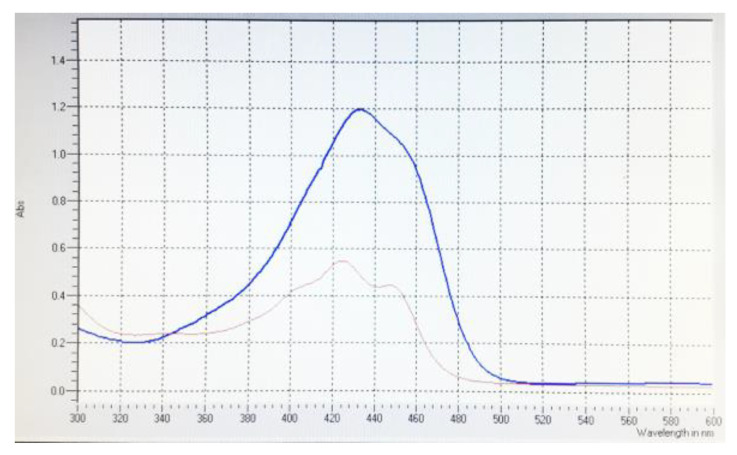
Curcumin (blue) and mitocurcumin (red) spectra at the same concentration (10 µM).

After the same treatment of MRSA ATCC 700698 strain with the studied compounds (100 µL of DMSO solutions—100 µM each in 4 mL liquid media with 5 × 10^5^ cells/mL), fluorescence microscopy revealed green stained cocci with a significant better brightness for mitocurcumin (Figure 2). As depicted in Figure 3, both compounds have fluorescent properties with similar spectra but with a more intense effect for curcumin.

### 2.3. Fluorescence Microscopy Studies Showed Reactive Species Accumulation for a S. pombe Eukaryotic Model

**Figure 4:**
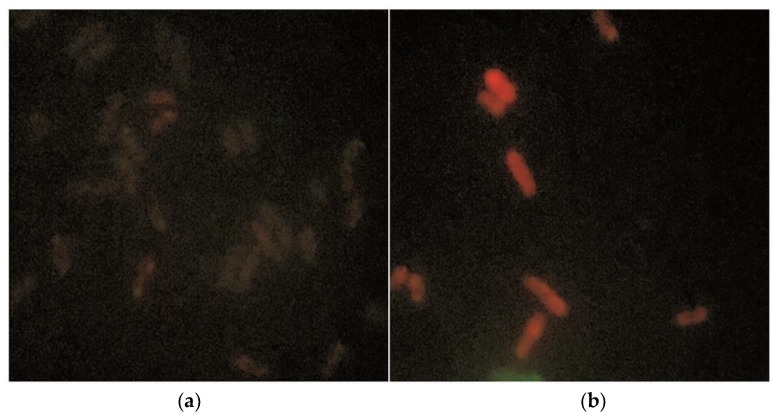
MitoTracker Red staining showed significant differences in the *S. pombe* MnSOD deleted eukaryotic model after mitocurcumin treatment. Both images use MitoTracker™ Red CM-H2Xros as ROS-sensitive mitochondria staining: (**a**) without any treatment and (**b**) after treatment with mitocurcumin 6 µM, 24 h.

After mitocurcumin treatment, the *S. pombe* cells showed significant modifications revealed by Mitotracker Red. Those modifications are more visible using the superoxide dismutase deleted strain, suggesting an intracellular accumulation of free radicals (Figure 4).

### 2.4. The Same Results Were Obtained with MitoTracker for the Prokaryotic Strains of MRSA (Figure 5)

The strain treated with mitocurcumin showed a significant difference of fluorescence intensity.

**Figure 5:**
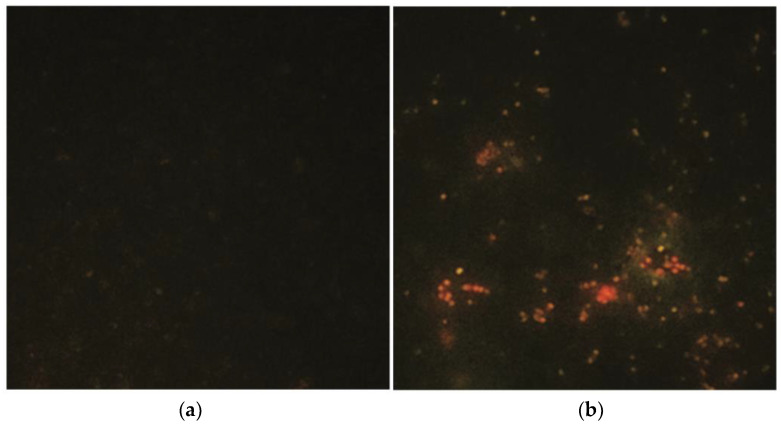
Fluorescence microscopy for an MRSA strain (ATCC 700698) stained with MitoTracker Red showed more intense intracellular production of ROS (**a**) after the mitocurcumin treatment compared (**b**) with no treatment.

### 2.5. The Antibacterial Activity of Mitocurcumin Is Modulated by Glutathione and Menadione

After the addition of reduced glutathione in culture media (antioxidant effect, 10 mM), the MIC obtained for MRSA strains increases to 3.9 µM (one doubling dilution of difference above) if compared with untreated strains (mitocurcumin MIC value of 1.95 µM). The opposite effect was obtained in the presence of menadione (prooxidant properties, 50 µM), when MIC of mitocurcumin decreases to 0.49 µM (two doubling dilution of difference below) (Table 3). We had no in-group variation. This modulatory effect is also preserved at different concentrations of glutathione and menadione (data not shown).

## 3. Discussion

Despite a large number of studies regarding the antibacterial effects of curcumin, most data suggest that a real benefit from this compound is hard to be obtained in vivo since the bioavailability of curcumin is poor and the concentrations needed are relatively high [25]. In comparison with curcumin, the triphenyl-phosphonium (TPP-) conjugate of curcumin has better stability and solubility.

The antibacterial effects of curcumin were extensively studied [12,26] but the triphenylphosphonium derivatives which are able to be used as antibiotics are relatively new [23,27,28], since the main purpose for those compounds was to target mitochondria in eukaryotic cells [20].

Considering the antibacterial activity, our study showed that mitocurcumin has a significantly higher effect than curcumin. While mitocurcumin displays strong antimicrobial efficacy against different strains of methicillin-resistant *Staphylococcus aureus* (MRSA) with an MIC value of 1.95 µM, curcumin showed an MIC value above 250 µM. Our observations with mitocurcumin are similar to the those published previously but with a subtle difference in MIC (MIC 1.6 µM). The MIC of curcumin against *S. aureus* has already been reported [12] and is significantly higher than in mitocurcumin. At the same time, the bioavailability of curcumin is a serious limiting factorin its potential use as antibiotic.

The first report of the antibacterial properties for mitocurcumin showed a wide range of values for this compound (including species of mycobacteria) and with better values of MIC for gram-positive cocci [23]. When compared with this study, our findings suggest a more important involvement of reactive oxygen species in the mechanism of action. One possible reason for the differences could rely on inappropriate choosing of the type and concentrations for the pro- and antioxidant agents.

Membrane-perturbing effects could also be responsible for the mechanism of the bactericidal action of triphenylphosphonium derivatives [29]. Alternatively, this may be attributed to bacterial bioenergetic suppression by way of collapsing membrane potential. Regarding this mechanism, it has been shown that SkQ1, a decyltriphenyl phosphonium cation conjugated to a quinone moiety, may perform protonophore-like action in combination with fatty acids [30]. Mitocurcumin is also reported to determine a rapid disruption of bacterial membrane potential [23]. When compared to conventional antibiotics, agents which target bacteria cell membranes are less likely to develop resistance [31].

On the other hand, as pro- or antioxidants, these compounds might be involved in ROS production/suppression [20]. Previous studies claimed that the intrabacterial prooxidant effect plays only a secondary role in the mitocurcumin antibacterial mechanism of action [23]. Our study suggests that this effect is more important than was previously believed, since the minimum inhibitory concentration was clearly modulated by both anti and prooxidant compounds. ROS generation can play a causal role in the bactericidal action for mitocurcumin. Experiments based on fluorescence microscopy clearly showed that exposure to MitoC leads to an increase in ROS levels in bacterial cells. Moreover, fluorescence microscopy studies showed reactive species accumulation for bacteria and for an *S. pombe* eukaryotic model. After mitocurcumin treatment, the *S. pombe* cells showed significant modifications revealed by MitoTracker Red. Those modifications seem to be more important for the superoxide dismutase deleted strain, suggesting an intracellular accumulation of free radicals.

The use of existing antibiotics can also determine metabolic stress, leading to the accumulation of NADH and the production of reactive oxygen species (ROS), which can quickly and completely disrupt vital processes [32]. Additionally, it was discovered that mitocurcumin can cause ROS production in cancer cells, causing apoptosis [33]. Experiments using DCF fluorescence showed that mitocurcumin exposure increased ROS levels in bacterial cells [23]. Antibacterial agents which target multiple mechanisms have been found to be more effective [34]. This concept, known as polypharmacology, is a common characteristic of effective drug use for cancer and other conditions [35].

Since the discovery of artemisinin [36], free radical implications in the mechanism of action for some chemotherapy drugs has been increasingly discussed. It was already demonstrated that mitocurcumin has strong antitumoral effects and the mechanism of action relies on free radical production at the mitochondrial level.

It is surprising that mitocurcumin lost its photochemical proprieties, which are specific to simple curcumin. A possible explanation for this is the disruption of electron conjugation in mitocurcumin due to the presence of two massive radicals which are able to delocalize the electrons. It is also noteworthy that the main antimicrobial mechanism of photoexcited curcumin involve intracellular reactive oxygen species (ROS) [37].

The stronger fluorescence effect on bacteria of mitocurcumin compared with simple curcumin suggests a better affinity of mitocurcumin for bacteria, since the spectral comparation shows a higher intensity for curcumin at the same concentrations (Figure 3).

A noteworthy outcome of the present study was the observation that mitocurcumin displays an MIC of 1.95 µM against methicillin-resistant *S. aureus* strains in vitro, pathogens which are considered by the WHO to be ‘priority pathogens’ that pose the greatest threat to human health [38] and most urgently require new treatments. The MIC value of mitocurcumin against drug-resistant *S. aureus* is approximately five times lower than the non-toxic concentration observed earlier for different types of human cells [23].

Various factors can impact a single MIC measurement, such as the components of the media, the density of the inoculum, the incubation conditions, and the setup of the test [39]. Individually, these factors may seem insignificant, but together they can contribute to the variability of the result. We avoided this variability by limiting the possible variation factors that can impact the MIC measurments. The results showed intergroup variations of one doubling dilution of difference above (for glutathione addition) and two doubling dilutions of difference below (for menadione addition) when compared to the mitocurcumin MIC value.

Despite its promising results in vitro, more research is needed to determine the full extent of mitocurcumin’s antimicrobial properties and its potential for use in clinical settings. As of now, the results of current studies suggest that mitocurcumin has the potential to be an effective and safe alternative to traditional antimicrobial agents, particularly in the treatment of multi-drug-resistant pathogens.

## 4. Materials and Methods

### 4.1. Bacterial Strains

Five strains of *Staphylococcus aureus* used in the current study were kindly provided by Dr. Aida Badescu from the Infectious Diseases Hospital of Iasi. Among these strains, 1 was methicillin-sensitive (ATCC 29213), 3 were MRSA hospital-tested strains (HS1, HS2, and HS3) and 1 was MRSA ATCC 700698.

### 4.2. Strains and Media for the S. pombe Model

The strain used in this work was a gift from Prof. Antony Carr (Genome Damage and Stability Center, Sussex University Brighton, UK) and was also the subject of manganese superoxide dismutase deletion [40].

Media used for *S. pombe* growth were as described [6]. Yeast cells were cultured at 30 °C in complete yeast extract plus supplements (YES) medium.

### 4.3. Maintenance and Preservation of Microorganisms

All the bacterial strains were grown on nutrient agar plates (Becton Dickinson, MD, USA) at 37 °C for 18 h. The cultures were stored at 4 °C and were re-cultured every 7 days.

### 4.4. Reagents

Curcumin, reduced glutathione, menadione, and dimethyl sulfoxide (DMSO) were obtained from Sigma Aldrich (St. Louis, MO, USA). Mitocurcumin (1,7-Bis{3-methoxy-4-[3-(triphenylphosphonium)propoxy]-phenyl} hepta-1,6-diene-3,5-dione dichloride) was purchased from Chiralsyn Laboratories, Hyderabad, India.

### 4.5. Preparation of Stock Solutions

The stock of mitocurcumin and curcumin solutions were prepared to a concentration of 5 mM/L using dimethyl sulfoxide as solvent. The final serial concentrations ranged from 250 to 0.39 µM by dilution with Mueller–Hinton broth media (Becton Dickinson, MD, USA).

Glutathione or menadione were added to the final solutions with concentrations ranging from 1 to 20 mM for glutathione and 1 to 100 µM for menadione.

### 4.6. Evaluation of Antimicrobial Activity

Single colonies on agar plates of 24 h were used to prepare the bacterial suspension. The optical density obtained must be between 0.08 and 0.1 which corresponds to a concentration of 10^7^–10^8^ CFU/mL according to McFarland. Turbidity of the bacterial suspension was measured at 600 nm. The microbial suspension was prepared in NaCl (0.9%).

Antibacterial capacities of mitocurcumin and curcumin were evaluated using the well diffusion method [41,42] and broth dilution assay [42,43,44]. For the well diffusion assay, 8 mm diameter wells were cut into a 5 mm Mueller–Hinton media after the cultivation of various strains of *S. aureus*. The wells were filled with 100 µL of solution with different concentrations of the tested compounds and the plates were incubated for 24 h at 37 °C. The inhibition zones were reported in millimeters (mm).

For the broth dilution assay, bacterial suspensions (5 × 10^5^ cells/mL) were incubated with 2-fold dilutions of DMSO solutions of tested compounds in 96-well non-binding surface plates for 24 h at 37 °C with mild shaking (250 rpm). The lowest concentration showing no visible growth was noted as the minimal inhibitory concentration (MIC).

All the experiments were undertaken in triplicate and repeated 3 times (n = 3).

### 4.7. Fluorescence Microscopy Studies

Since the curcumin and mitocurcumin alone exhibit fluorescence at 365 nm, an Olympus BX51 fluorescence microscope (Japan) was used to study the compound accumulation in bacteria. MitoTracker™ Red CM-H2Xros (a mitochondrial ROS-sensitive fluorescent dye) was the selected choice for *S. pombe* microscopy fluorescence studies of mitochondria [45,46] and for the bacterial cells [45]. Briefly, cells were separated by centrifugation, resuspended in phosphate-buffered saline (PBS) with pH 7.4, and incubated for 15 min at 37 °C with 50 μg/mL MitoTracker Red CM-H2XRos.

## 5. Conclusions

The efficient antibiotic activity against MRSA strains, as assessed by in vitro studies, suggests mitocurcumin as a highly promising lead molecule. As mechanism of antibacterial action, ROS generation can play a causal role in bactericidal action for mitocurcumin, since the minimum inhibitory concentration was clearly modulated by both anti and prooxidant compounds and fluorescence microscopy showed a more significant intracellular accumulation of free radicals both in bacteria and in the eukaryotic model of *S. pombe*.

## Figures and Tables

**Table 1:** Inhibition diameters of curcumin and mitocurcumin (both at 10 µM) for MRSA.

Strain	Inhibition Diameter mm (Curcumin)	Inhibition Diameter mm (Mitocurcumin)
MRSA strain (ATCC 700698)	0	17 ± 1

**Table 2:** Minimal inhibitory concentrations of curcumin and mitocurcumin for different MRSA.

Strain	MIC—µM (Curcumin)	MIC—µM (Mitocurcumin)
MRSA strain (ATCC 700698)	>250	1.95
HS1 *	>250	1.95
HS2 *	>250	1.95
HS3 *	>250	1.95
ATCC 29213	>250	1.95

* MRSA hospital tested strains (HS1, HS2, and HS3).

**Table 3:** The influence of reduced glutathione and menadione for the antibacterial activity of mitocurcumin.

Strain	MIC—µM (mitocurcumin)	MIC—µM (mitocurcumin + glutathione)	MIC—µM (mitocurcumin + menadione)
MRSA strain (ATCC 700698)	1.95	3.9	0.49

## Data Availability

Not applicable.
